# Paroxysmal Supraventricular Tachycardia and Troponin Elevation: Insights into Mechanisms, Risk Factors, and Outcomes

**DOI:** 10.3390/jcm14165644

**Published:** 2025-08-09

**Authors:** Georgios Aletras, Emmanuel Koutalas, Maria Bachlitzanaki, Maria Stratinaki, Irene Bachlitzanaki, Spyridon Stavratis, Gerasimos Garidas, Michael Pitarokoilis, Emmanuel Foukarakis

**Affiliations:** 1 Department of Cardiology, Venizelio General Hospital of Heraklion, 71409 Heraklion, Greece; maria.stratinaki@gmail.com (M.S.); eirinibach@outlook.com.gr (I.B.); spirostavratis@gmail.com (S.S.); gerasimosgaridas@yahoo.gr (G.G.); michpit@gmail.com (M.P.); mfouk@hotmail.com (E.F.); 2 School of Medicine, University of Crete, 70013 Heraklion, Greece; medp2011922@med.uoc.gr; 3 Department of Cardiology, University Hospital of Heraklion, 71500 Heraklion, Greece; emmkout@gmail.com; 4 Second Department of Internal Medicine, Venizelio General Hospital of Heraklion, 71409 Heraklion, Greece

**Keywords:** paroxysmal supraventricular tachycardia, mechanisms, risk factors, myocardial injury, coronary artery disease, outcomes

## Abstract

**Background:** Elevated cardiac troponin (cTn) levels in patients with paroxysmal supraventricular tachycardia (PSVT) often prompt coronary artery disease evaluation, though the clinical relevance of this finding remains unclear. This study aimed to identify risk factors for cTn elevation after a PSVT episode and assess its clinical significance, including the role of coronary artery disease (CAD) and long-term outcomes. **Methods:** We retrospectively collected data on demographics, presenting symptoms, comorbidities, chronic antiarrhythmic medication use, tachycardia duration, admission systolic blood pressure (SBP), heart rate (HR), laboratory findings, and cardioversion method in patients presenting to the Emergency Department (ED) with PSVT over a 4-year period. Patients were stratified into two groups based on the presence or absence of troponin elevation. Individuals with elevated cTn levels and at least one cardiovascular risk factor were further evaluated for CAD. One-year outcomes included SVT recurrence, rehospitalization, ablation, and mortality. **Results:** Among 120 patients with PSVT, 58 (48.3%) exhibited elevated cardiac troponin (cTn) levels. Independent predictors of cTn elevation included retrosternal chest pain, absence of prior SVT history, higher admission HR, and lower SBP. A heart rate cut-off of 165 bpm was identified as optimal for predicting cTn elevation (sensitivity 62.1%, specificity 72.6%). Of the 58 cTn (+) patients, 25 underwent CAD evaluation, with only 1 case (4%) confirming significant coronary disease. At one-year follow-up (n = 118), troponin elevation was not associated with increased SVT recurrence, rehospitalization, ablation, or mortality. Similarly, CAD evaluation in troponin-positive patients did not predict outcomes. **Conclusions:** Troponin elevation after PSVT is frequent but not prognostically significant. It is likely due to transient myocardial stress rather than CAD, supporting a conservative, individualized approach to further testing.

## 1. Introduction

The 2019 European Society of Cardiology (ESC) Guidelines for the management of patients with supraventricular tachycardia defined supraventricular tachycardia (SVT) as an umbrella term, which encompasses tachycardias (atrial and/or ventricular rates in excess of 100 beats per minute at rest), with mechanisms originating at or above the His bundle [1]. This group includes inappropriate sinus tachycardia, focal or multifocal atrial tachycardia [AT], macroreentrant AT such as typical atrial flutter, junctional tachycardia, atrioventricular nodal reentrant tachycardia (AVNRT), and various forms of accessory pathway-mediated reentrant tachycardias like atrioventricular reentrant tachycardia (AVRT). Paroxysmal supraventricular tachycardia (PSVT) represents a subset of SVT and is defined as a clinical syndrome characterized by the presence of a regular tachycardia of abrupt onset and termination, most commonly caused by AVNRT, AVRT, or AT [2]. The prevalence of PSVT is estimated at 3.5% with a consistent female predominance and increasing incidence with age [2,3].

Palpitations are the most common symptom of PSVT, followed by chest pain, dyspnea, and presyncope or syncope. In most cases, sinus rhythm is rapidly restored using vagal maneuvers (e.g., carotid sinus massage, Valsalva maneuver) or intravenous adenosine administration, allowing for early discharge from the Emergency Department (ED). Although PSVT generally has a favorable prognosis, its clinical presentation can mimic acute coronary syndrome (ACS), particularly when accompanied by chest pain, dyspnea, and possible electrocardiographic changes during arrhythmia. In such cases, clinicians may measure cardiac biomarkers, including high-sensitivity cardiac troponin I or troponin T (hs-cTnI/hs-cTnT), although routine troponin testing in PSVT remains controversial [4,5].

Cardiac troponins (cTnI/cTnT) are the biomarkers of choice for detecting myocardial injury and play a key role in diagnosing myocardial infarction (MI). In MI, troponin levels rise within hours and remain elevated for several days [6]. However, troponin release is not exclusive to MI, as multiple mechanisms—including apoptosis, transient imbalance of oxygen supply and demand, cellular release of proteolytic degradation products of troponin, myocardial stretching, inflammation, and normal myocyte turnover—can lead to troponin elevation without irreversible myocardial necrosis [7,8].

In cardiomyocytes, most cTn is bound to tropomyosin, while approximately 8% is unbound and present in the cytoplasm, commonly referred to as the “cytosolic pool” [9]. Following myocardial injury, the release of cTn in blood exhibits a biphasic pattern. In the first phase, cytosolic troponin is released due to increased membrane permeability, while the second phase reflects the breakdown of myofibrillar-bound troponin, with a prolonged release lasting up to 5–7 days. Some studies suggest that troponin may be released without permanent cell necrosis, as cytoplasmic proteins may be released through membrane blebs, which contain cytosolic troponin and lyse upon entering the bloodstream. This mechanism may explain how troponin circulates in the blood without cell death [8,10,11].

Beyond obstructive coronary artery disease (Type 1 MI), troponin elevation can result from both cardiac and non-cardiac causes, such as myocardial injury due to oxygen supply–demand mismatch (Type 2 MI), heart failure, myocarditis, or systemic conditions like sepsis and chronic kidney disease [6]. Tachyarrhythmias, including PSVT, often lead to elevated troponin levels, which may represent a non-specific response secondary to tachycardia rather than obstructive coronary artery disease (CAD). Despite this, patients with elevated troponin values during PSVT often undergo further CAD evaluation, potentially leading to unnecessary invasive testing.

This study aimed to identify predictors of troponin elevation after a PSVT episode and evaluate its association with underlying CAD. One-year clinical outcomes—including SVT recurrence, rehospitalization, ablation, and mortality—were also assessed in relation to troponin status and coronary evaluation.

## 2. Materials and Methods

This was a retrospective, single-center observational study conducted at Venizelio General Hospital of Heraklion, the second largest hospital on the island of Crete, with a total capacity of 431 beds and approximately 100,000 annual Emergency Department (ED) visits. The study period spanned from 1 July 2020 to 1 September 2024. This study was reviewed and approved by the hospital’s Research and Ethics Committee.

### 2.1. Inclusion Criteria

Eligible participants were adult patients (≥18 years) presenting to the ED with paroxysmal supraventricular tachycardia (PSVT), including atrioventricular nodal reentrant tachycardia (AVNRT), atrioventricular reentrant tachycardia (AVRT), and atrial tachycardia (AT), with symptom onset within 24 h.

### 2.2. Exclusion Criteria

Patients were excluded from this study based on the following criteria:

▪ Insufficient data available in the medical records.
▪ History of structural heart disease, including the following:
  ○ Cardiomyopathy;
  ○ Severe left ventricular hypertrophy;
  ○ Significant valvular disease (moderate or severe stenosis or regurgitation);
  ○ Left ventricular ejection fraction below 50% as documented by an echocardiogram performed within the past year or a bedside echocardiogram following cardioversion.

▪ Chronically elevated troponin values.
▪ Diagnosis of COVID-19 at presentation. During the study period, all patients admitted to the Cardiology Department were screened with rapid antigen tests for COVID-19, regardless of symptoms. PCR testing was selectively performed in patients with flu-like symptoms, elevated inflammatory markers, or known exposure to confirmed cases. Patients with a confirmed COVID-19 infection by either method were excluded from the study. No IgG or IgM serologic testing was performed, and a history of prior asymptomatic infection was not routinely available.
▪ Presence of other potential underlying causes of PSVT, such as pulmonary embolism, infection, myocarditis, etc. Myocarditis was excluded based on clinical and anamnestic criteria. Patients with symptoms suggestive of viral illness (e.g., fever, myalgia), elevated inflammatory markers, electrocardiographic or echocardiographic abnormalities indicative of myocardial inflammation, or recent exposure to confirmed COVID-19 cases underwent further evaluation, including PCR testing. No additional imaging (e.g., cardiac magnetic resonance imaging) or serologic testing was routinely performed.

### 2.3. Methodology

Data were extracted from ED records and electronic medical files. We retrieved basic demographic characteristics (sex, age), main symptoms (1 or more) such as retrosternal chest pain, palpitations, syncope/presyncope, and gastrointestinal discomfort and underlying comorbidities such as arterial hypertension (HTN), diabetes mellitus (DM), dyslipidemia, thyroid disease, known coronary artery disease (CAD), autoimmune disease, and previous history of supraventricular tachycardia (SVT). A history of CAD was defined as previous myocardial infarction, abnormal non-invasive stress test finding, or CAD by invasive coronary angiography (including a history of percutaneous coronary intervention).

Data on the duration of symptoms, antiarrhythmic medications (e.g., beta-blockers, calcium-channel blockers, class IC antiarrhythmics, and amiodarone), vital signs on admission (systolic blood pressure, heart rate), and laboratory parameters (e.g., high-sensitivity cardiac troponin I, creatinine, and urea) were recorded. It is noted that some patients were on one or more antiarrhythmic medications (e.g., b-blocker plus class IC). Further details on cardioversion type (e.g., spontaneous, vagal maneuvers, beta-blockers, calcium channel blockers [CCBs], intravenous adenosine, and electrical cardioversion) and patient admissions to the Cardiology Department were documented.

Patients were categorized according to whether or not their troponin levels exceeded the 99th percentile threshold (Group One: elevated cTn values—cTn (+), Group Two: normal hs-cTnI values—cTn (−)). Elevated cTn values were defined as any cTn measurement above the 99th percentile upper reference limit (URL). Siemens Healthineers High-Sensitivity Troponin I assays were used, and values were reported as multiples above or below the sex-specific 99th percentile upper reference limit (URL), which is 53.5 pg/mL for men and 38.6 pg/mL for women, according to the manufacturer. The assessment of acute coronary syndrome (ACS) risk followed the 2020 ESC Guidelines for the management of NSTE-ACS [12]. High-sensitivity cardiac troponin I (hs-cTnI) testing was performed based on clinical discretion and institutional protocols. Although the 0–1 h and 0–2 h diagnostic algorithms are recommended by the guidelines for rapid rule-out of ACS, these were not implemented in our settingdue to prevailing local clinical practice during the study period. Instead, a 0–3 h approach was predominantly used when repeat testing was clinically indicated. Specifically, in patients presenting with retrosternal chest pain, electrocardiographic abnormalities, or symptom onset within six hours, a second troponin measurement was obtained three hours after the initial one, and the higher value was recorded. Otherwise, the initial measurement was sufficient. Overall, 96 out of 120 patients (80%) underwent serial troponin measurements, while 24 patients (20%) had only a single measurement—15 in the cTn (−) group and 9 in the cTn (+) group.

Coronary evaluation was performed mostly at the discretion of the treating cardiologist, but typically in patients with elevated troponin levels, at least one cardiovascular risk factor, and no prior coronary artery disease (CAD) evaluation within the previous two years. All patients who underwent CAD evaluation fulfilled these criteria. The decision to proceed with either invasive coronary angiography or coronary CT angiography (CCTA) was based on multiple factors, including the patient’s cardiovascular risk profile, presence of chest pain or ECG abnormalities, recent history of CAD evaluation, echocardiographic findings, and patient preference.

Significant CAD was defined based on invasive coronary angiography findings as the presence of >50% stenosis in the left main stem, >70% stenosis in a major coronary vessel, or intermediate stenosis (30% to 70%) with a fractional flow reserve (FFR) below 0.8. If coronary computed tomography angiography (CCTA) revealed suspicious findings, patients underwent invasive coronary angiography for further evaluation. If CCTA was normal, no further testing was performed due to its high negative predictive value.

Follow-up data were retrieved through electronic medical records. Outcomes assessed within one year of the index episode included all-cause mortality, rehospitalization for any reason, Emergency Department visits due to PSVT recurrence, and whether the patient underwent catheter ablation. One-year follow-up data were available for 118 out of 120 patients (98.3%): 57 patients in the cTn (+) group and 61 in the cTn (−) group. The remaining two patients were excluded from the 1-year analysis because their index PSVT episode occurred close to the end of the study period, and the 1-year follow-up mark had not yet been reached by the time of data collection.

### 2.4. Statistical Analysis

Data analysis was performed using IBM SPSS Statistics for Windows, Version 29.0.2.0, Armonk, NY, USA: IBM Corp. Continuous variables were tested for normality using the Kolmogorov–Smirnov test and p-p plots. Normally distributed quantitative variables were presented as mean ± standard deviation, while non-normally distributed variables were presented as median and interquartile range (IQR). Categorical variables were presented as frequencies and percentages. Comparisons of normally distributed continuous variables were performed using Student’s *t*-test, while non-normally distributed variables were analyzed using the Mann–Whitney *U* test. Associations between categorical variables were assessed using the chi-square test. Multivariate logistic regression analysis was conducted to identify independent predictors of troponin elevation following PSVT episodes. Receiver operating characteristic (ROC) curves were generated, and the area under the curve (AUC) was calculated for the heart rate, using the Youden index to determine the optimal cut-off value for elevated troponin values. A *p*-value < 0.05 was considered statistically significant.

## 3. Results

### 3.1. General Characteristics

A total of 147 patients presented to the ED with PSVT during the four-year study period, of whom 120 met the inclusion criteria and were thus included in the analysis.

**Figure 1:**
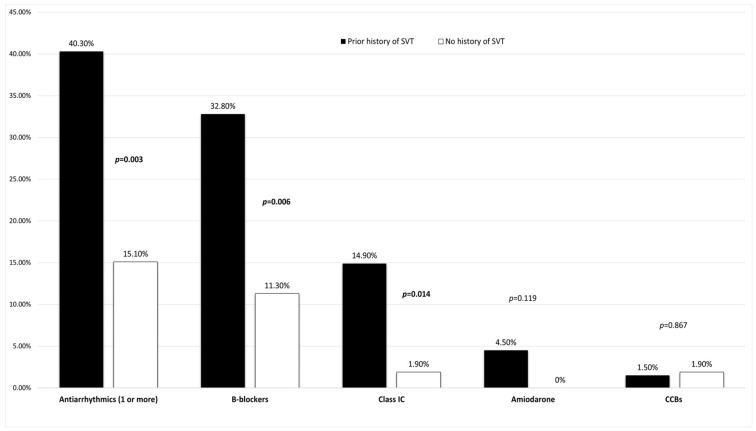
Comparison of antiarrhythmic medications in patients with and without a prior history of SVT. SVT: supraventricular tachycardia; CCBs: calcium channel blockers. Bold values indicate statistical significance (*p* < 0.05).

The mean age was 57 years, with a predominance of women (63.3%). Palpitations were the most common symptom (95%), followed by retrosternal chest pain (13.3%). Regarding comorbidities, known CAD (7.5%), diabetes mellitus (11.7%), hypertension (30.8%), or thyroid disorders (20%) were observed in a minority of patients, whereas a previously known history of SVT (55.8%) was the most frequent comorbidity. Approximately 30% of patients were already on antiarrhythmic medication, with a statistically significant proportion having a previous history of SVT (40.3% with SVT history vs. 15.1% without, *p* = 0.003) (Table 1, Figure 1).

Patients with a history of SVT exhibited higher systolic blood pressure values (133 ± 19 mmHg vs. 127 ± 21 mmHg, *p* = 0.042) on admission compared to those without previous episodes. The median duration of tachycardia was two hours, with a mean systolic blood pressure of 130 mmHg and a median heart rate of 160 beats per minute (bpm) at presentation. Intravenous adenosine was the most frequently used cardioversion method (69.8%), followed by vagal maneuvers (12.26%). Spontaneous cardioversion, beta-blockers, calcium channel blockers, and electrical cardioversion were rarely utilized. The median hs-cTnI value was 0.88 times below the upper normal limit (UNL), and renal function abnormalities were rare (Table 1). Information regarding prior COVID-19 infection was not systematically collected and thus not available for analysis.

### 3.2. Risk Factors for Troponin Elevation

Among the 120 patients, 58 (48.33%) had elevated high-sensitivity cardiac troponin I values (hs-cTnI), with a median hs-cTnI level 4.28 times the UNL. A statistically significant difference between the two groups was observed regarding retrosternal pain, SVT history, admission systolic blood pressure, and heart rate. Specifically, patients with elevated cTn values more often reported retrosternal pain (22.4% vs. 4.8%, *p* = 0.005) and had a higher admission heart rate (172.5 bpm vs. 150 bpm, *p* < 0.001) and lower systolic blood pressure values (126 ± 16 vs. 136 ± 22, *p* = 0.007). Antiarrhythmic medication use was significantly associated with lower heart rate on admission (150 bpm vs. 165 bpm, *p* = 0.011) (Table 2). Furthermore, patients with a known history of SVT were more likely to have normal hs-cTnI values (44.8% vs. 66.1%, *p* = 0.019). No statistically significant differences between the two groups were found regarding other comorbidities, e.g., DM, CAD, duration of tachycardia, or cardioversion type (Table 1). A subgroup analysis by sex revealed no statistically significant difference in troponin elevation or key hemodynamic parameters. However, women had significantly lower serum creatinine values and a lower prevalence of diabetes mellitus. Full details are presented in Appendix A.1, Table A1.

Among the 58 patients with elevated hs-cTnI values, 5 (8.6%) were initially admitted with a working diagnosis of non-ST-elevation acute coronary syndrome (NSTE-ACS). However, obstructive CAD was not confirmed following coronary angiography, and all five patients were ultimately discharged with PSVT-related diagnoses as no further work-up was deemed necessary.

Multivariate logistic regression analysis identified retrosternal chest pain, absence of a prior history of SVT, admission heart rate, and systolic blood pressure as independent risk factors of elevated cTn values after an episode of PSVT (Table 3).

**Figure 2:**
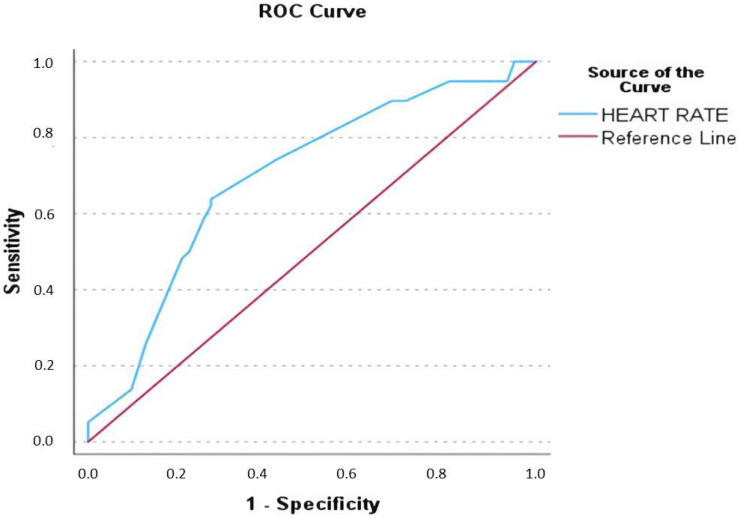
Receiver operating characteristic (ROC) curve analysis for determining the admission heart rate cut-off to predict elevated cardiac troponin levels. The analysis yielded an area under the curve (AUC) of 0.697. A heart rate of 165 beats per minute (bpm) was identified as the optimal cut-off, with a sensitivity of 62.1% and a specificity of 72.6% for its association with elevated troponin levels.

Receiver operating characteristic (ROC) curve analysis for admission heart rate yielded an area under the curve (AUC) of 0.697, with a heart rate of 165 bpm identified as the optimal cut-off (62.1% sensitivity, 72.6% specificity) for its association with elevated cTn values (Figure 2).

### 3.3. Coronary Artery Disease Evaluation

A total of 51 patients were admitted to the Cardiology Department, of whom 41 (80.4%) had elevated hs-cTnI levels. Among these, 25 underwent further evaluation for coronary artery disease (CAD), while the remaining 16 patients did not, due to a recent (within two years) CAD evaluation (n = 3), absence of cardiovascular risk factors (n = 4), patient refusal (n = 2), or loss to follow-up (n = 7). Among the 25 evaluated patients, 14 underwent invasive coronary angiography, while 11 underwent coronary computed tomography angiography (CCTA). Significant coronary artery disease was identified in only one patient (4%). In these cases, additional diagnostic testing (e.g., cardiac magnetic resonance imaging or positron emission tomography) for non-coronary causes of troponin elevation was not performed, as the troponin rise was attributed to tachycardia-induced Type 2 myocardial infarction, and there were no clinical or imaging findings suggestive of alternative myocardial pathology.

### 3.4. Outcomes Within One Year According to Troponin Status

Among the 118 patients with complete one-year follow-up data, SVT recurrence occurred in 28 individuals (23.7%). There was no statistically significant difference between patients with elevated troponin levels and those with normal values (21% vs. 26.2%, *p* = 0.508), and an exploratory comparison of baseline characteristics between patients with and without SVT recurrence also revealed no statistically significant differences (Appendix A.2, Table A2).

Rehospitalization for any cause was observed in 13 patients (11.0%), including 5 (8.7%) in the cTn (+) group and 8 (13.1%) in the cTn (−) group (*p* = 0.451).

Ablation procedures were performed in 24 patients (20.3%) during follow-up, with a numerically higher rate in the cTn (+) group compared to cTn (−) (24.5% vs. 16.3%, respectively), though the difference was not statistically significant (*p* = 0.273).

Only one death (0.8%) was recorded during the one-year follow-up, occurring in the cTn (−) group (1.6%), with no deaths in the cTn (+) group (*p* = 0.336).

### 3.5. Impact of CAD Evaluation on Clinical Outcomes in cTn (+) Patients

Among the 57 patients with elevated troponin levels and available follow-up data, 25 (43.9%) underwent further evaluation for coronary artery disease (CAD). One-year outcomes did not significantly differ between those who underwent CAD evaluation and those who did not. SVT recurrence occurred in 16% of patients who underwent CAD evaluation versus 25% of those who did not (*p* = 0.303), rehospitalization in 12% vs. 6.3% (*p* = 0.528), and ablation in 24% vs. 25% (*p* = 0.750), respectively. No deaths were recorded in either group (*p* = 1.000). These findings suggest no significant prognostic differences associated with CAD evaluation among troponin-positive patients during the first year (Table 4).

## 4. Discussion

This study found that nearly half of patients presenting with PSVT had elevated troponin levels. Independent predictors of troponin elevation included retrosternal chest pain, absence of a prior SVT history, higher admission heart rate, and lower systolic blood pressure. Despite the frequent troponin elevation, obstructive CAD was rarely found, and there were no prognostic implications during one-year follow-up.

Paroxysmal supraventricular tachycardia (PSVT) is a common and usually benign arrhythmia. However, it can mimic acute coronary syndromes, making clinical management complex, particularly when elevated cTn levels or electrocardiographic abnormalities are present [13]. In our study, nearly 50% of patients experienced elevated hs-cTnI levels following a PSVT episode, which is consistent with prior studies by Yedder et al. (32.5%) and Bukkapatnam et al. (48%), highlighting the frequent occurrence of elevated cTn in this population [14,15]. This study expands upon previous findings by identifying independent predictors of troponin elevation in PSVT and examining their potential clinical implications.

Our cohort demonstrated a predominance of female patients (63.3%), which is consistent with epidemiological data. Risk factors for CAD and ACS, including diabetes mellitus, hypertension, and prior CAD, were less prevalent in our study population compared to other atrial arrhythmias such as atrial fibrillation [2,4]. The most common cardioversion methods were intravenous adenosine and vagal maneuvers, with no significant association between cardioversion type and troponin elevation. This aligns with prior findings that adenosine, with reported success rates of 78–96% in terminating PSVT, is both safe and effective [2].

### 4.1. Possible Mechanisms of Troponin Elevation

The precise mechanism of tachycardia-induced troponin elevation remains unclear. The most widely accepted hypothesis suggests that increased myocardial oxygen demand combined with reduced coronary perfusion during tachycardia leads to transient ischemia and subsequent cTnI release [4,13]. Our findings support this hypothesis, as higher admission heart rates and lower systolic blood pressure were independently associated with troponin elevation. This is consistent with prior studies linking tachycardia severity to troponin release [15,16].

Other possible but less well-studied mechanisms in tachycardia-induced myocardial injury include increased sarcolemmal permeability following cardiomyocyte stress, allowing cTn fragments from an early releasable pool to leak into the bloodstream. Stressors such as contraction, beta-adrenergic stimulation, myocardial stretching, or transient ischemia may increase the rate of this release. Similarly, an increased myocardial turnover or apoptosis may also lead to transient troponin elevation, similar to findings in endurance exercise studies [17].

In our study, elevated cTn was associated with retrosternal chest pain, higher admission heart rate, lower systolic blood pressure, and the absence of a prior history of SVT. While chest pain is a subjective symptom with significant interindividual variability, its strong association with troponin elevation suggests that it may serve as an early indicator of myocardial stress in PSVT, which is consistent with findings by Sayadnik et al. [16,18].

A higher heart rate likely shortens diastolic filling time, thereby impairing myocardial perfusion and leading to ischemia and troponin release. Similarly, lower systolic blood pressure at admission may reflect hemodynamic compromise due to tachycardia-induced reduction in cardiac output, further contributing to troponin release. These findings align with previous studies by Sayadnik et al. and Yedder et al., which also identified heart rate as a key determinant of troponin elevation [15,16]. It should be noted that no direct hormonal or coronary vasoreactivity assessments were performed. As such, our proposed mechanisms remain hypothesis-generating. Future prospective studies, including stress biomarker analysis and coronary flow testing (e.g., coronary flow reserve), are needed to validate these mechanisms. 

### 4.2. Absence of SVT History: A Marker of Susceptibility to Troponin Release

A novel and intriguing finding of our study was that the absence of a prior history of supraventricular tachycardia (SVT) emerged as an independent risk factor for troponin elevation, a relationship that has been scarcely explored in the existing literature [4].

One possible explanation for this is the concept of cardiac adaptation, particularly in patients with frequent recurrences. Recurrent tachycardia episodes may lead to altered autonomic regulation, improved energy utilization, and possibly an increased ischemic threshold over time. This concept parallels findings in endurance athletes, where experienced runners demonstrated lower post-exercise troponin elevations compared to less-trained individuals [17,19,20]. We hypothesize that patients without prior SVT episodes may lack such myocardial conditioning, rendering them more susceptible to transient ischemia and troponin release during an acute episode, a hypothesis warranting further investigation [17].

Furthermore, the acute psychological stress associated with a first tachycardia episode may also contribute to myocardial injury. The initial episode often represents a significant emotional and physiological stressor, triggering autonomic and neuroendocrine responses via the autonomic nervous system (ANS) and the hypothalamic–pituitary–adrenal (HPA) axis. These responses can provoke hemodynamic, inflammatory, and metabolic alterations that predispose patients to myocardial ischemia, analogous to exercise stress-induced ischemia but unrelated to coronary obstruction severity or previous revascularization [21].

Acute stress leads to increased sympathetic activation, with β1-adrenergic stimulation promoting tachycardia and myocardial contractility, while α1-adrenergic stimulation induces vasoconstriction. Alterations in coronary artery diameter during mental stress have been found to parallel the effects observed with intracoronary acetylcholine infusion. This response may reflect coronary microvascular endothelial dysfunction, which can lead to a reduction in wall shear stress (WSS) within upstream epicardial arteries, thereby promoting the formation of focal epicardial atherosclerotic lesions [21,22].

Acute mental stress also triggers peripheral microvascular constriction and stimulates endothelial and smooth muscle cells in the tunica media via α-adrenergic stimulation, leading to systemic vasoconstriction and elevated blood pressure. Unlike physical exertion, mental stress does not prompt increased blood flow to large muscle groups, resulting in reduced peripheral perfusion. This mismatch results in higher left ventricular afterload and increased myocardial oxygen demand [21].

Mental stress-induced myocardial ischemia (MSIMI) is notably more prevalent in women, particularly postmenopausal women. In the study by Mehta et al., women with ischemia and no obstructive coronary artery disease (INOCA) exhibited greater peripheral microvascular constriction in response to mental stress compared to age-matched asymptomatic controls [22]. Takotsubo syndrome, often considered the extreme manifestation of MSIMI, predominantly affects postmenopausal women following physical or emotional stressors. These stressors trigger a surge of catecholamines, leading to multivessel coronary vasospasm and coronary microvascular dysfunction, which contribute to acute myocardial ischemia and heart failure [23,24].

Given that our study cohort was predominantly female (63.3%) with a mean age of 57 years, it is plausible that sympathetic hyperactivity and coronary microvascular dysfunction may have contributed to troponin elevation in some patients, consistent with mechanisms described in MSIMI and Takotsubo syndrome [21,23]. However, our sex-based subgroup analysis did not demonstrate significant differences in troponin elevation, clinical presentation, or CAD evaluation between men and women. These findings suggest that while stress-related myocardial ischemia remains a plausible mechanism in select patients, particularly women, our study was not designed or powered to detect subtle sex-specific pathophysiological differences. Further prospective studies focused on sex hormones, stress reactivity, and coronary microvascular function are warranted.

### 4.3. The Role of Antiarrhythmic Medication

Although antiarrhythmic medication was not significantly associated with troponin elevation in our study, its higher use among patients with a history of SVT may play a preventive role by reducing the frequency or severity of tachycardic episodes. Antiarrhythmics, particularly beta-blockers, could help blunt the extreme hemodynamic effects of tachycardia, potentially lowering myocardial oxygen demand and reducing the risk of troponin release. Beta-blockers decrease heart rate, allowing for longer diastolic filling time and improved coronary perfusion, thereby reducing ischemic burden. In our study, patients on antiarrhythmic medication presented with lower heart rates, reflecting this indirect protective effect on myocardial injury, which may also account for the lower systolic blood pressure observed in patients without a history of SVT [25].

### 4.4. Clinical Implications and CAD Evaluation

Despite widespread troponin elevation, our findings reinforce that the prevalence of obstructive CAD in PSVT patients is low. In our study, only 4% of patients undergoing further evaluation were diagnosed with significant CAD, which is consistent with prior studies by Yedder et al. (5.26%) and Enbergs et al. (7.3%) [15,26]. These findings challenge the necessity of routine CAD testing in PSVT patients with troponin elevation. While electrocardiographic changes alone are not predictive of CAD [14], comprehensive clinical evaluation, including patient history, symptoms, troponin kinetics, and echocardiographic findings, should guide further testing. Suspicious echocardiographic findings, such as newly identified regional wall motion abnormalities or the presence of multiple cardiovascular risk factors, may warrant selective CAD evaluation [15].

It is also important to note that coronary evaluation was selectively performed only in patients with elevated hs-cTnI levels. The rate of coronary imaging in this group was 43.1%, while none of the hs-cTnI-negative patients underwent further coronary testing. The choice between invasive angiography and non-invasive CCTA was determined by clinical context and resource availability, emphasizing the need for individualized decision-making. These findings underscore the importance of tailoring CAD work-up in PSVT patients based on risk assessment, rather than relying on troponin elevation alone.

### 4.5. One-Year Follow-Up

In our study, one-year clinical outcomes—including SVT recurrence, all-cause rehospitalization, ablation, and mortality—did not significantly differ between patients with and without elevated troponin levels nor among troponin-positive patients based on whether they underwent CAD evaluation. These findings align with previous PSVT-specific studies, which also reported no association between troponin positivity and short-term adverse events [4,5,27]. Notably, in our cohort, only one death was recorded, and recurrence and rehospitalization rates were low and comparable across groups. While studies involving broader tachyarrhythmia populations have suggested a potential prognostic role for troponin—particularly in patients with atrial fibrillation or known CAD—this signal does not appear to extend to low-risk PSVT patients without structural heart disease [5].

These findings are further supported by a recent multicenter study by Chen et al., which evaluated elderly patients with PSVT and found no significant difference in 5-year major adverse cardiovascular events (MACEs) between troponin-positive and troponin-negative groups. Importantly, the only independent predictor of MACEs in that study was a history of coronary artery disease, suggesting that troponin elevation in isolation does not predict long-term outcomes in this population [28]. Similarly, Bandorski et al. found that in patients with SVT or ventricular tachycardia (VT), repeated troponin I measurements could aid in CAD detection but were not specific for distinguishing arrhythmia type. Their study emphasizes that while elevated troponin may point toward ischemia, it must be interpreted cautiously in the absence of corroborating evidence [29].

A recent meta-analysis by Pourmand et al. similarly concluded that troponin elevation in SVT patients has limited utility in predicting major adverse cardiovascular events (MACEs), with only 11% of troponin-positive cases experiencing MACEs during long-term follow-up. Importantly, the pooled data highlighted that most troponin-positive patients underwent further cardiac testing, yet only a small fraction derived clear benefit. Moreover, elevated troponin alone was not predictive of adverse outcomes unless accompanied by established cardiac risk factors, such as known CAD [5].

Collectively, these findings suggest that in hemodynamically stable PSVT patients without significant comorbidities, isolated troponin elevation should be interpreted with caution. Troponin measurement may increase hospital admissions and resource utilization without improving outcomes. Therefore, in the absence of chest pain, ischemic ECG changes, or known cardiovascular disease, clinicians should consider omitting routine troponin testing in PSVT and rely instead on clinical judgment and validated decision tools such as the HEART score [4,5,27].

### 4.6. Limitations

Our study has several limitations, primarily due to its retrospective nature, which limited the accuracy and completeness of data collection, particularly regarding potential confounding factors such as smoking status, obesity, family history of cardiovascular disease, more detailed echocardiographic parameters, and electrocardiographic signs.

The retrospective design also limited the availability of certain data points. Information regarding prior COVID-19 infection was not recorded, and no serologic testing (IgG/IgM) was performed. Although all patients underwent rapid antigen testing upon presentation, and selected cases received confirmatory PCR testing, the possibility of asymptomatic prior infection cannot be entirely excluded. In addition, advanced imaging (e.g., cardiac magnetic resonance imaging) was not routinely performed in troponin-positive patients without obstructive CAD, as such modalities were not widely available and often required out-of-pocket costs. Their use was typically reserved for diagnostically uncertain cases. As a result, alternative causes of troponin elevation, such as myocarditis, could not be definitely ruled out.

Not all patients underwent serial troponin testing, which may have led to underestimation of troponin elevation rates. This reflects both the retrospective nature of our study and the current clinical practice, in which serial biomarker assessments are not routinely performed unless clinically indicated. Additionally, the sample size was small, drawn from a single center, which may limit the generalizability of the findings. We acknowledge that this reflects the exploratory nature of this study. Future multicenter collaborations with larger and more diverse populations are needed to validate these findings and improve external validity.

Moreover, the predominance of female patients may have influenced certain stress-related mechanisms, such as susceptibility to mental stress-induced myocardial ischemia. Although the study cohort had a high proportion of female patients, a post hoc sex-based subgroup analysis was conducted and is presented in Section 3 and Appendix A.1, Table A1. This analysis did not reveal significant sex-based differences in troponin elevation. However, further studies are warranted to explore potential gender-specific mechanisms underlying PSVT-related myocardial injury.

Finally, the symptoms, comorbidities, and duration of tachycardia were based on patient recall and medical records, which can introduce bias and result in either overestimation or underestimation of the true impact of confounding factors on the occurrence of elevated cTn values.

## 5. Conclusions

Elevated cTn values are common among patients with paroxysmal supraventricular tachycardia (PSVT). Our findings indicate that the presence of retrosternal chest pain, higher admission heart rate, lower SBP, and the absence of a prior history of SVT are independent risk factors for elevated troponin levels following a PSVT episode. However, the elevation is more likely due to a mismatch between myocardial oxygen supply and demand rather than the presence of coronary artery disease (CAD). While mechanisms such as myocardial stress, increased sarcolemmal permeability, or stress-related sympathetic activation were discussed, these conclusions are not safe to be expressed based on the present data and require further prospective validation.

From a prognostic standpoint, elevated troponin levels were not associated with increased rates of SVT recurrence, any-cause rehospitalization, or mortality during one-year follow-up. These findings suggest that troponin elevation alone does not indicate a worse clinical outcome in otherwise stable PSVT patients and should be interpreted within the broader clinical context.

Based on our results, the decision to pursue CAD evaluation following PSVT should be individualized. Clinical history, comorbidities, troponin kinetics, and echocardiographic findings should guide further testing rather than relying solely on troponin elevation. Prospective studies are needed to clarify which patients may benefit from additional evaluation and which can be safely discharged after successful PSVT termination in Emergency Departments.

## Figures and Tables

**Table 1:** Demographics, comorbidities, main symptoms, chronic medication, cardioversion type, initial laboratory evaluation, and patient outcomes within a year. Bold values indicate statistical significance (*p* < 0.05) * SVT: supraventricular tachycardia, ^+^ UNL: upper normal limit.

Characteristics	Total (n = 120)	Elevated Troponin (n = 58)	Normal Troponin (n = 62)	*p*-Value
**Demographics:**				
Male sex	44 (36.7%)	20 (34.5%)	24 (38.7%)	0.631
Age (years)	57 ± 14	56 ± 12	57 ± 16	0.796
**Symptoms:**				
Palpitations	114 (95%)	54 (93.1%)	60 (96.8%)	0.357
Chest pain	16 (13.3%)	13 (22.4%)	3 (4.8%)	**0.005**
Syncope/presyncope	7 (5.8%)	5 (8.6%)	2 (3.2%)	0.208
Gastrointestinal tract disturbance	3 (2.5%)	2 (3.4%)	1 (1.6%)	0.520
**History and medication:**				
History of SVT *	67 (55.8%)	26 (44.8%)	41 (66.1%)	**0.019**
Diabetes mellitus	14 (11.7%)	7 (12.1%)	7 (11.3%)	0.894
Hypertension	37 (30.8%)	15 (25.9%)	22 (35.5%)	0.254
Known coronary artery disease	9 (7.5%)	5 (8.6%)	4 (6.5%)	0.652
Thyroid disorders	24 (20%)	14 (24.1%)	10 (16.1%)	0.273
Dyslipidemia	52 (43.3%)	28 (48.3%)	24 (38.7%)	0.291
Autoimmune disease	7 (5.8%)	4 (6.9%)	3 (4.8%)	0.631
**Antiarrhythmics** **(1 or more)**	35 (29.2%)	16 (27.6%)	19 (30.6%)	0.713
B-blockers	28 (23.3%)	13 (22.4%)	15 (24.2%)	0.818
Calcium channel blockers	2 (1.7%)	2 (3.4%)	0 (0%)	0.140
Class IC	11 (9.2%)	2 (3.4%)	9 (14.5%)	0.056
Amiodarone	3 (2.5%)	1 (1.7%)	2 (3.2%)	0.599
**Duration of tachycardia and vital signs:**				
Duration of tachycardia (hours)	2 (1–6)	3 (1–6)	2 (1–4.25)	0.195
Admission heart rate (beats per minute)	160 (150–180)	172.5 (150–190)	150 (140–170)	**<0.001**
Admission systolic blood pressure (mmHg)	130 ± 20	126 ± 16	136 ± 22	**0.007**
**Cardioversion type:**				
Vagal maneuvers	13 (10.8%)	8 (13.8%)	5 (8.1%)	0.313
Intravenous adenosine	83 (69.2%)	44 (75.9%)	39 (62.9%)	0.125
B-blockers/class IC	8 (6.7%)	2 (3.4%)	6 (9.7%)	0.172
Spontaneous	10 (8.3%)	3 (5.2%)	7 (11.3%)	0.226
Electrical	4 (3.3%)	1 (1.7%)	3 (4.8%)	0.342
Calcium channel blockers	2 (1.7%)	0 (0%)	2 (3.2%)	0.168
**Admission laboratory parameters:**				
High-sensitivity cardiac troponin I (multiples above or below for UNL ^+^)	0.88 (0.23–4.11)	4.28 (2.15–13.33)	0.24 (0.1–0.48)	**<0.001**
Creatinine (mg/dL)	0.9 (0.78–1.06)	0.92 (0.79–1.08)	0.88 (0.76–1.06)	0.390
Urea (mg/dL)	35 (28–45)	35 (27–46)	35 (28.5–43.5)	0.838
In-hospital admission	51 (42.5%)	41 (70.7%)	10 (16.1%)	**<0.001**
**Outcomes within a year (n = 118):**				
SVT recurrence	28 (23.7%)	12 (21%)	16 (26.2%)	0.508
Rehospitalization (any cause)	13 (11%)	5 (8.7%)	8 (13.1%)	0.451
Ablation	24 (20.3%)	14 (24.5%)	10 (16.3%)	0.273
Death	1 (0.8%)	0 (0%)	1 (1.6%)	0.336

**Table 2:** Effect of antiarrhythmic medication on parameters associated with elevated troponin values. Bold values indicate statistical significance (*p* < 0.05) * SVT: supraventricular tachycardia; ^+^ BP: blood pressure.

Parameters	Antiarrhythmics (+) [n = 35]	Antiarrhythmics (−) [n = 85]	*p*-Value
Heart rate (beats per minute)	150 (135–180)	165 (150–180)	**0.011**
Retrosternal chest pain	5 (14.3%)	11 (12.9%)	0.844
History of SVT *	27 (77.1%)	40 (47.1%)	**0.003**
Systolic BP ^+^ (mmHg)	130 (110–141)	130 (120–145)	0.924

**Table 3:** Multivariable analysis for predicting independent risk factors for elevated troponin values after an episode of paroxysmal SVT. Bold values indicate statistical significance (*p* < 0.05) SVT: supraventricular tachycardia; ^+^95% CI: 95% Confidence Interval; +OR: Odds Ratio.

	Univariate			Multivariate		
Parameters	+OR	^+^95% CI	*p*	+OR	^+^95% CI	*p*
Retrosternal chest pain	5.681	1.527–21.140	0.01	4.761	1.123–20.190	**0.034**
History of SVT	0.416	0.199–0.870	0.020	0.431	0.187–0.994	**0.048**
Heart rate (beats per minute)	1.030	1.013–1.048	0.001	1.030	1.012–1.049	**0.001**
Systolic blood pressure (mmHg)	0.974	0.955–0.994	0.01	0.977	0.956–0.999	**0.042**

**Table 4:** One-year clinical outcomes in troponin-positive patients according to coronary artery disease evaluation. * SVT: supraventricular tachycardia; ^+^ CAD: coronary artery disease.

Outcome:	CAD ^+^ Evaluation (+) (n = 25)	CAD ^+^ Evaluation (−) (n = 32)	*p*-Value
SVT * recurrence	4 (16%)	8 (25%)	0.303
Rehospitalization (all-cause)	3 (12%)	2 (6.3%)	0.528
Ablation	6 (24%)	8 (25%)	0.750
Death	0 (0%)	0 (0%)	1.000

## Data Availability

Due to ethical constraints and the need to protect patient confidentiality, the datasets generated and analyzed during the current study are not publicly available. However, data may be made available from the corresponding author upon reasonable request and with approval from the Bioethics and Ethics Committee of Venizelio General Hospital of Heraklion.

## Abbreviations

The following abbreviations are used in this manuscript:

95% CI	95% confidence interval
ACS	Acute coronary syndrome
ANS	Autonomic nervous system
AT	Atrial tachycardia
AUC	Area under the curve
AVNRT	Atrioventricular nodal reentrant tachycardia
AVRT	Atrioventricular reentrant tachycardia
BPM	Beats per minute
CAD	Coronary artery disease
CCBs	Calcium channel blockers
CCTA	Coronary computed tomography angiography
cTn	Cardiac troponin
CVD	Cardiovascular disease
DM	Diabetes mellitus
ED	Emergency department
ESC	European Society of Cardiology
Hs-cTnI/T	High-sensitivity cardiac troponin I/T
HPA	Hypothalamic pituitary adrenal
HR	Heart rate
HTN	Hypertension
INOCA	Ischemia with no obstructive coronary artery disease
LVEF	Left ventricular ejection fraction
MACEs	Major adverse cardiovascular events
MI	Myocardial infarction
MSIMI	Mental stress-induced myocardial infarction
NSTE-ACS	Non-ST-elevation acute coronary syndrome
OR	Odds ratio
PSVT	Paroxysmal supraventricular tachycardia
ROC	Receiver operating curve
SBP	Systolic blood pressure
VT	Ventricular tachycardia
UNL	Upper normal limit
URL	Upper reference limit
WSS	Wall shear stress

## Appendix A

### Appendix A.1. Comparison of Clinical Characteristics Between Male and Female Patients

As shown in Appendix A.1, Table A1, no statistically significant difference was observed between sexes regarding troponin elevation, vital signs, or primary symptoms such as palpitations and chest pain. However, female patients had significantly lower serum creatinine levels (*p* < 0.001) and a lower prevalence of diabetes mellitus (*p* = 0.023). These findings are consistent with known sex-related differences in cardiovascular risk profiles [30] but did not translate into significant differences in myocardial injury as measured by hs-cTnI.

**Table A1:** Comparison of baseline clinical, hemodynamic, and laboratory characteristics between male and female patients with paroxysmal supraventricular tachycardia. Bold values indicate statistical significance (*p* < 0.05) ^#^ bpm: beats per minute; ^++^ CAD: coronary artery disease; * SVT: supraventricular tachycardia; and ^+^ UNL: upper normal limit.

Parameter	Male (n = 44)	Female (n = 76)	*p*-Value
Age (years)	60.5 (51–66)	54 (47–69)	0.336
**Symptoms:**			
Palpitations	40 (90.9%)	74 (97.4%)	0.118
Chest pain	6 (13.6%)	10 (13.2%)	0.941
Syncope/presyncope	3 (6.8%)	4 (5.3%)	0.726
Gastrointestinal disturbance	3 (6.8%)	0 (0%)	**0.021**
**History and medication:**			
History of SVT *	23 (52.3%)	44 (57.9%)	0.550
Diabetes mellitus	9 (20.5%)	5 (6.6%)	**0.023**
Hypertension	17 (38.6%)	20 (26.3%)	0.159
Known coronary artery disease	4 (9.1%)	5 (6.6%)	0.615
Thyroid disorders	7 (15.9%)	17 (22.4%)	0.394
Dyslipidemia	23 (52.3%)	29 (38.2%)	0.133
Autoimmune disease	1 (2.3%)	6 (7.9%)	0.205
**Antiarrhythmics (1 or more)**			
B-blockers	10 (22.7%)	18 (23.7%)	0.905
Calcium channel blockers	1 (2.3%)	1 (1.3%)	0.693
Class IC	3 (6.8%)	8 (10.5%)	0.498
Amiodarone	1 (2.3%)	2 (2.6%)	0.903
**Duration of tachycardia and vital signs:**			
Duration of tachycardia (hours)	2 (1–6)	3 (1–6)	0.078
Admission heart rate (bpm ^#^)	160 (150–180)	160 (141–180)	0.171
Admission systolic blood pressure (mmHg)	130 (115–140)	130 (120–150)	0.599
**Admission laboratory parameters:**			
High-sensitivity cardiac troponin I (multiples above or below for UNL ^+^)	0.7 (0.15–3.11)	0.99 (0.25–4.64)	0.575
Creatinine (mg/dL)	1.05 (0.9–1.28)	0.8 (0.7–0.95)	**<0.001**
Urea (mg/dL)	38 (30–47.5)	34 (27–43)	0.062
**Hospital admission**	18 (40.9%)	33 (43.4%)	0.789
**CAD ^++^ evaluation**	8 (18.2%)	17 (22.3%)	0.658

### Appendix A.2. Analysis of Potential Predictors of SVT Recurrence

The assessment of possible risk factors for SVT recurrence during the first year was based on comparisons of baseline characteristics, comorbidities, medication use, and admission parameters between patients with and without recurrence. As shown in Table A2, no statistically significant differences were observed between the two groups.

**Table A2:** Comparison of demographic, clinical, and admission variables between patients who experienced SVT recurrence and those who did not during the first year of follow-up. No statistically significant differences were observed between the groups. * SVT: supraventricular tachycardia; ^+^ UNL: upper normal limit; ^#^ bpm: beats per minute; and ^@^ Hs-cTnI: high-sensitivity cardiac troponin I.

Parameter	SVT *-Recurrence (+) (n = 28)	SVT *-Recurrence (−) (n = 90)	*p*-Value
Age (years)	59 ± 15	56 ± 13	0.217
Male sex	9 (32.1%)	35 (38.8%)	0.570
**Symptoms:**			
Palpitations	26 (92.8%)	86 (95.5%)	0.692
Chest pain	4 (14.3%)	12 (13.3%)	0.866
Syncope/presyncope	1 (3.6%)	6 (6.7%)	0.560
Gastrointestinal disturbance	1 (3.6%)	2 (2.2%)	0.678
**History and medication**			
History of SVT *	17 (60.7%)	50 (55.5%)	0.553
Diabetes mellitus	3 (10.7%)	11 (12.2%)	0.858
Hypertension	10 (35.7%)	27 (30%)	0.523
Known coronary artery disease	1 (3.6%)	8 (8.8%)	0.367
Thyroid disorders	3 (10.7%)	21 (23.3%)	0.161
Dyslipidemia	13 (46.4%)	39 (43.3%)	0.706
Autoimmune disease	0 (0%)	7 (7.7%)	0.133
**Antiarrhythmics on admission (1 or more):**			
B-blockers	6 (21.4%)	22 (24.4%)	0.785
Calcium channel blockers	0 (0%)	2 (2.2%)	0.431
Class IC	4 (14.3%)	7 (7.7%)	0.284
Amiodarone	0 (0%)	3 (3.3%)	0.333
**Duration of tachycardia and vital signs:**			
Duration of tachycardia (hours)	3 (1–6)	2 (1–6)	0.531
Admission heart rate (bpm ^#^)	150 (142–80)	160 (150–180)	0.361
Admission systolic blood pressure (mmHg)	130 (120–150)	130 (119–140)	0.524
Hs-cTn-I ^@^ (multiples above or below for UNL ^+^)	0.85 (0.14–2.34)	0.94 (0.24–5.8)	0.322
